# Development of Real-Time Monitoring System Based on IoT Technology for Curing Compound Application Process during Cement Concrete Pavement Construction

**DOI:** 10.3390/s23198187

**Published:** 2023-09-30

**Authors:** Soon Ho Baek, Kang In Lee, Seong-Min Kim

**Affiliations:** Department of Civil Engineering, Kyung Hee University, Gyeonggi 17104, Republic of Korea; qortnsgh1@khu.ac.kr (S.H.B.); leerkddls123@khu.ac.kr (K.I.L.)

**Keywords:** cement concrete pavement, curing compound, flowmeter, image processing, Internet of Things, monitoring, sensing

## Abstract

Among the construction processes of Portland cement concrete pavement (PCCP), the curing compound spraying process is one of the most important processes. If the curing compound spraying amount does not meet the standard or if the curing compound is not applied evenly, distresses occur at the early age of construction, ultimately causing deterioration in concrete pavement performance. The purpose of this study is to develop a real-time monitoring system for a curing compound spraying process based on the Internet of Things (IoT) and sensing technologies to improve the construction quality of concrete pavement. To achieve the goal of this research, we conducted various laboratory and field experiments. The curing compound spraying amount and sprayed status were measured and analyzed using flowmeters, image acquisition sensors, and an image processing program, and the data were provided to workers in real time and simultaneously transmitted to the IoT cloud to form a database. From this study, it is confirmed that the IoT-technology-based curing compound spraying amount and sprayed status monitoring systems can be successfully established to manage construction quality related to the curing of concrete pavement.

## 1. Introduction

Like other structures, construction management is very important for Portland cement concrete pavement (PCCP) to ensure excellent long-term performance. The construction process of PCCP is divided into concrete pouring work, surface smoothness work, and surface treatment work such as tinning, curing compound spraying work, and joint cutting work. Among these construction processes, the curing compound spraying process can be judged as a simple task, but it is one of the most important processes for securing long-term performance of PCCP. The curing of cement concrete pavement is a process to prevent the loss of strength by suppressing rapid moisture evaporation in the process of concrete hardening and hydration. In the case of cement concrete pavement construction, a membrane curing method in which a curing compound is sprayed on the surface of the pavement is generally used.

**Figure 1:**
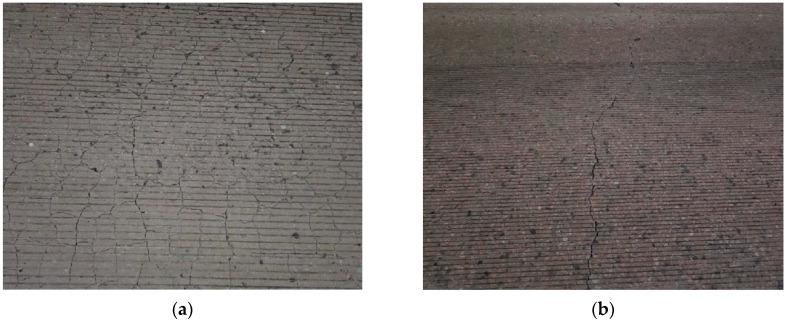
Early-age distresses in PCCP: (**a**) plastic shrinkage cracks and (**b**) transverse crack.

Each country or local government applies the standard value of the curing compound spraying amount as shown in Table 1 in consideration of the local environmental conditions and the characteristics of the construction methods [1,2,3,4,5,6,7,8,9,10,11,12,13,14,15,16,17,18]. When spraying the curing compound, the amount of the curing compound that meets the standard should be evenly sprayed on the surface of the concrete pavement. If the curing compound spraying amount does not meet the standard or if the curing compound is not applied evenly, distresses such as surface plastic shrinkage cracks, longitudinal cracks, or transverse cracks occur at the early age of construction as shown in Figure 1, ultimately causing deterioration in concrete pavement performance. It is noted that if the concrete pavement is not properly cured, the actual strength is lower than the design strength, and moisture evaporation from the pavement surface is significant. In these cases, the distresses mentioned earlier occur.

Because the curing of concrete pavement is very important, research on it has been continuously conducted. Studies were conducted on the general aspects of concrete pavement curing [19,20] and were also conducted to evaluate the curing status of concrete pavement [21,22,23,24,25,26,27]. The effective curing thickness concept was introduced as a method to evaluate the effectiveness of a curing method [21]. The concrete pavement curing evaluation methods using non-destructive testing were developed [22,23]. A new curing effectiveness evaluation protocol was introduced, consisting of using measured relative humidity and temperature to calculate an effectiveness index [24]. The viability of identified curing quality indicators was reviewed [25]. Research was conducted to understand the effects of curing compounds and their application methods on concrete properties [26]. A laboratory-based procedure, which bridges laboratory-measured parameters to performance in the field, was developed as part of a process to qualify the effectiveness of a curing compound membrane [27]. Research on curing compound materials and applications have also been conducted [28,29,30,31,32]. The effect of different curing compound applications on the behavior of field pavement was assessed [28]. The effectiveness of curing compounds on concrete pavement was studied using different curing compound materials [29,30,31,32].

By combining the Internet of Things (IoT) and sensing technologies, pavement construction quality can be improved based on scientific evaluation. Recently, such research works have been conducted in the pavement field and suggest future directions [33,34,35,36]. However, most technologies and research related to smart construction of pavements have been applied to asphalt concrete pavements [37,38,39,40,41,42,43,44,45,46,47,48], and limited research has been conducted on smart construction technologies for cement concrete pavements [49,50,51,52]. In particular, research related to curing compound spraying is extremely limited [53].

The purpose of this study is to develop a real-time monitoring system for a curing compound spraying process based on IoT and sensing technologies to improve the construction quality of concrete pavement and ultimately ensure excellent long-term pavement performance. When managing a curing compound spraying process, both the curing compound spraying amount and sprayed status must be considered. Managing the curing compound spraying amount is to monitor whether the curing compound amount that meets the standards is being sprayed, and managing the curing compound sprayed status is to monitor whether the curing compound is evenly sprayed on the concrete pavement surface. Even if the total amount of curing compound sprayed meets the standards, if the curing compound is not applied evenly due to problems such as clogging of the spray nozzle when spraying the curing compound, a decrease in pavement performance due to curing problems may occur in areas where the curing compound is insufficiently sprayed. Although the curing process is very important, a scientific method to evaluate if the curing compound is properly sprayed has not been available. In this study, the curing compound spraying amount and sprayed status are measured and analyzed using flowmeters and image sensors, and the data are provided to workers in real time and simultaneously transmitted to the IoT cloud to form a database. Thereby, a monitoring system is established to manage construction quality related to the curing of concrete pavement. The details and results of this study are presented in this paper.

## 2. Development of Monitoring System of Curing Compound Spraying Amount

In order to develop a monitoring system of the curing compound spraying amount, first, a flowmeter capable of properly measuring the flow rate of the curing compound was selected, and the applicability was identified by verifying the accuracy of the selected flowmeter. Then, a system was developed where it could evaluate the adequacy of the curing compound spraying amount by attaching the flowmeter to the curing compound spraying equipment and transmit data related to the curing compound spraying amount in real time.

### 2.1. Selection of Flow Measurement Sensor

To determine the capacity of the flowmeter that can be used for actual field measurement, an analysis was conducted by applying the Korean Construction Standards for curing compound spraying on cement concrete pavements, which is about 0.45 L/m^2^ [1,2]. Since the spraying amount of the curing compound should be varied according to the moving speed of the curing compound spraying equipment, the actual moving speed of the curing compound spraying equipment was investigated by visiting the concrete pavement construction site. As a result of the investigation, the curing compound spraying equipment moved a distance of 6 m, which is the joint interval of the jointed concrete pavement, in about 12 s, and therefore the moving speed was analyzed to be about 0.5 m/s. Based on the field survey, the spraying amount of the curing compound while moving the curing compound spraying equipment can be calculated as in Equation (1), considering the moving speed of the equipment, the width of the pavement, and the standard for the amount of curing compound per unit area. As a result, the curing compound spraying rate was suitable for the standard when about 2 L is sprayed per second.

Curing compound spraying rate (L/s) =Equipment speed (m/s) × Pavement width (m) × Standard spraying amount (L/m^2^) =0.5 × 9.0 × 0.45 = 2.025 (L/s)(1)

**Figure 2:**
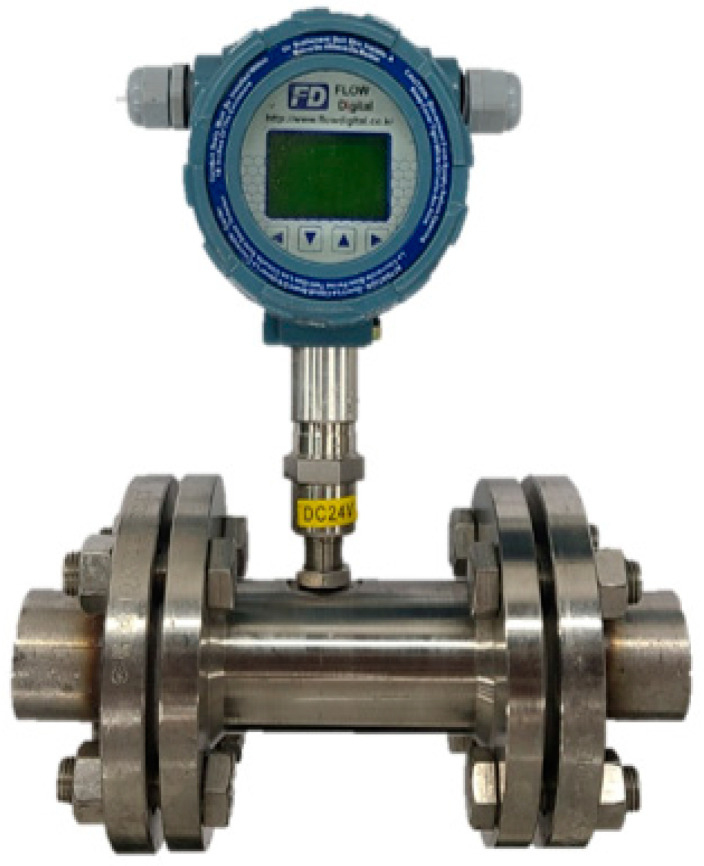
Assembly containing selected turbine flowmeter.

Finally, the sensor for measuring the flow rate was selected, Flow Digital’s turbine flowmeter FD_TFM model shown in Figure 2, which could measure the flow rate between 0.417 and 4.17 L/s, which is up to twice the standard curing compound spraying rate when using the pipe with a diameter of 32 mm [54]. In addition, it is convenient to attach and detach from the spraying equipment, and real-time data transmission is easy. It is noted that the curing compound spraying machines may have different sizes of supply pipes. In this case, the flowmeters with different pipe sizes can be used, or an extension pipe fitting both the flowmeter and the supply pipe at both ends can be used with a sufficient length to reduce flow turbulence caused by changes in pipe diameter.

### 2.2. Accuracy Analysis of Flowmeter

**Figure 3:**
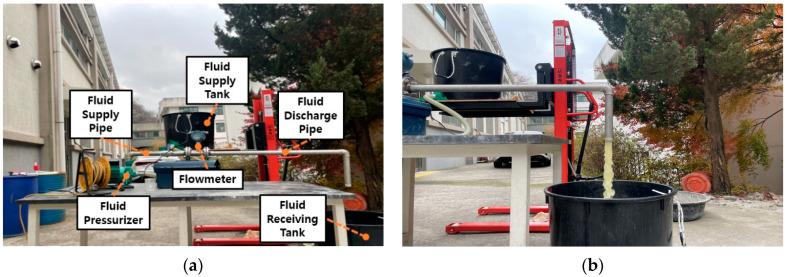
Laboratory experiment of fluid flow measurement: (**a**) experimental system and (**b**) experimental process.

A fundamental experiment was conducted to determine whether the curing compound spraying amount could be accurately measured by installing the selected turbine flowmeter on the curing compound spraying equipment. As shown in Figure 3, the experimental system consisted of a fluid supply tank, a fluid pressurizer, a fluid supply pipe, a flowmeter, a fluid discharge pipe, and a fluid receiving tank. The fluid contained in the fluid supply tank is supplied through the fluid supply pipe at a constant pressure using the fluid pressurizer, and the flow rate was measured when the fluid passed through the flowmeter. The fluid passing through the flowmeter was contained in the fluid-receiving tank through the fluid discharge pipe.

The experiment was conducted to analyze the accuracy of the flowmeter according to the range of flow rate by adjusting the range of flow rate in four different stages using the water and curing compound. Since the turbine flowmeter measures the flow rate while the turbine rotates when the pipe is filled with more than 2/3 of the fluid, the experiment was conducted while the fluid was initially discharged, and the pipe was filled with fluid. In order to analyze the usability and accuracy of the flowmeter, the average measurement value of the fluid volume measured for 10 s and the weight (mass) of the actual fluid passing through the flowmeter were compared. In the case of water, the specific weight was 1, so the water volume and mass can be considered the same. However, since the specific weight of curing compound is smaller than that of water, the measured mass of the curing compound should be compared with the curing compound volume measured using the flowmeter, multiplying the specific weight of the curing compound. For reference, the specific weight of the curing compound used in this study was 0.86 g/mL.

Table 2 and Table 3 show the test results of the accuracy analysis of the flowmeter using the water and curing compound, respectively. Measurements were made at four different flow rates, and as a result of experiments using water, the accuracy of the flowmeter was over 96%. In the case of the curing compound, it can also be seen that the measured volume using the flowmeter and the volume contained in the fluid-receiving tank are quite similar even when the flow rate is changed, and even in this case, the average accuracy was higher than 96%. In the case of using the curing compound, as already mentioned, the results were obtained in consideration of the specific weight of the curing compound. Therefore, it was evaluated that there would not be any problem in measuring the spraying amount of the curing compound by applying this monitoring system. It is noted that because there were some changes in flow rate throughout the test and there were some errors when receiving fluid using a fluid-receiving tank, the accuracy obtained in this test is expected to be sufficient for use in the field.

### 2.3. Algorithm of Curing Compound Spraying Amount Monitoring System

**Figure 4:**
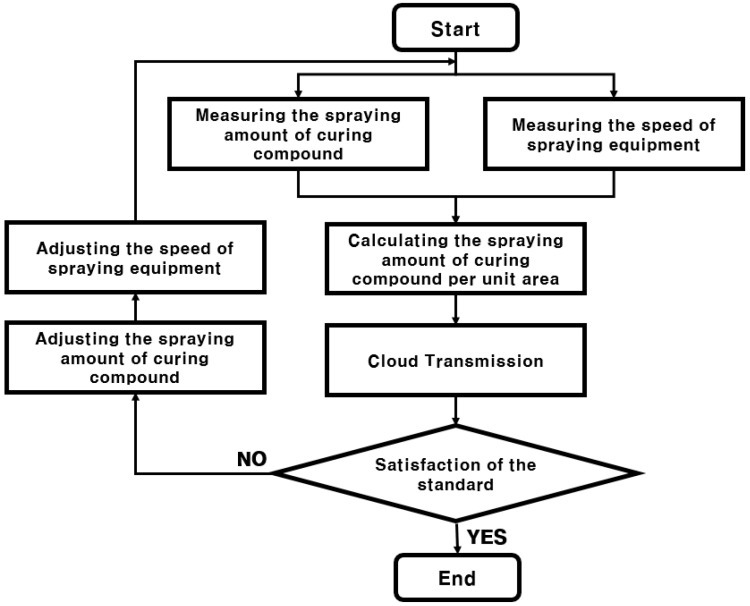
Algorithm of curing compound spraying amount monitoring system.

The algorithm of the curing compound spraying amount monitoring system is shown in Figure 4. First, the curing compound spraying amount is measured using a flowmeter, and at the same time, the moving distance of the curing compound spraying equipment is measured using GPS data. Then, the curing compound spraying amount per unit area is calculated as shown in Equation (2) using the measured spraying amount of curing compound, moving distance of the equipment, and concrete pavement width. It is noted that the curing compound spraying amount per unit area is the spraying amount of curing compound divided by the sprayed area that is the moving distance of the spraying equipment multiplied by the concrete pavement width.

Curing compound spraying amount per unit area (L/m^2^) =Curing compound spraying amount (L)/(Moving distance of spraying equipment (m) × Pavement width (m))(2)

In the curing compound spraying amount monitoring system, various measured and calculated values are transmitted to the IoT cloud and stored. These types of data include construction time, spraying equipment speed, equipment’s latitude, longitude and altitude, curing compound spraying rate, curing compound spraying amount, and curing compound spraying amount per unit area. Through the cloud, it is possible to promote quality control by providing measurement and analysis data to construction personnel in real time. If it is evaluated that the curing compound spraying amount does not meet the standard, the curing compound spraying process can be calibrated in real time by adjusting the curing compound spraying amount or by adjusting the speed of the curing compound spraying equipment.

## 3. Development of Monitoring System of Curing Compound Sprayed Status

In order to develop a curing compound sprayed status monitoring system, an image acquisition sensor capable of properly photographing the curing compound sprayed status was first selected, and a program for analyzing the image of the curing compound sprayed status was developed. In addition, we selected the acceptance criteria for the analyzed image of the sprayed status. Then, a system was developed that could evaluate the adequacy of the curing compound sprayed status by attaching the image acquisition sensor to the curing compound spraying equipment and transmitting data related to the curing compound sprayed status in real time.

### 3.1. Selection of Image Acquisition Sensor

**Figure 5:**
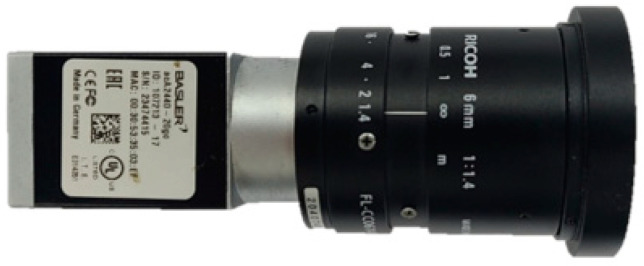
Assembly of selected image acquisition sensor and lens.

The image acquisition sensor for analyzing the curing compound sprayed status must first be able to interconnect the IP between the image acquisition sensor and the personal computer, the output value of the sensor should be over 20 fps for the analysis of the sprayed status, and it should be an industrial image acquisition sensor that can utilize gigabit Ethernet (GigE) to make image processing more efficient. Therefore, in this study, Basler’s acA2440-20gc image acquisition sensor and Ricoh’s FL-CC0614A-2M lens were selected as shown in Figure 5, and they all satisfied the selection criteria [55,56].

### 3.2. Image Analysis Program of Curing Compound Sprayed Status

**Figure 6:**
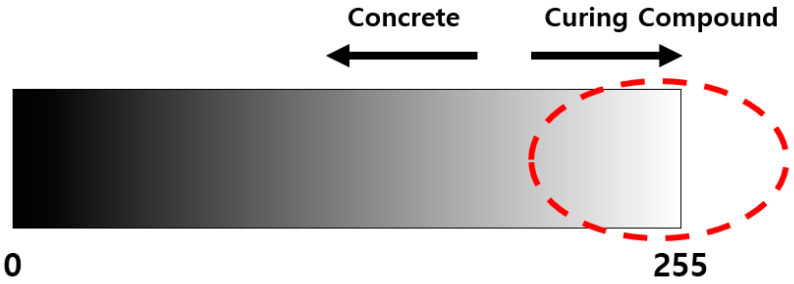
Grayscale image analysis.

In the curing work of cement concrete pavement construction, the white curing compound is mainly sprayed. Therefore, the areas where the curing compound is properly applied to the surface of the concrete pavement show a white color, while the areas where the curing compound is applied inappropriately show a gray color, which is the color of the concrete. With this difference in color, it can be confirmed whether the curing compound is sprayed evenly. With this in mind, a method for evaluating the curing compound sprayed status is developed using image processing techniques. The analysis method can be divided into analysis using RGB and analysis using grayscale. Compared to grayscale, color analysis using RGB is more diverse in color classification, so analysis processing time is longer, and it is not easy to select a reference value. Therefore, in this study, an image processing method using grayscale is selected, which can be quickly analyzed at the construction site and is easy to select a reference value. Grayscale, as shown in Figure 6, ranges from 0 to 255, and the closer to 0, the blacker the color, and the closer to 255, the whiter the color.

**Figure 7:**
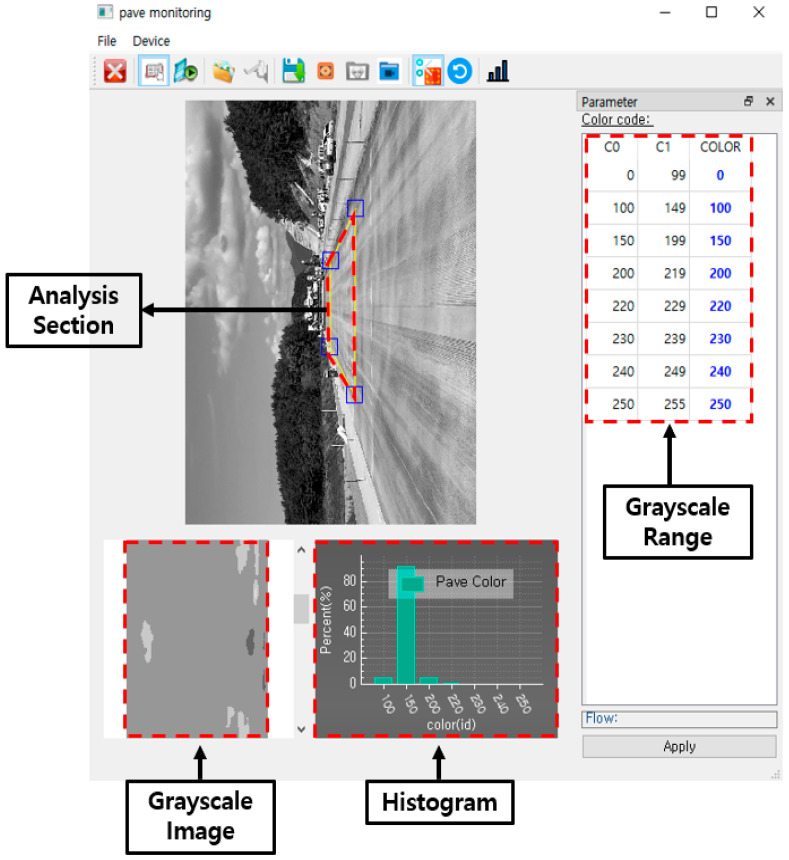
Image processing program.

In this study, in order to evaluate whether the curing compound is sprayed evenly, an image processing program was developed, as shown in Figure 7, that can convert the image of the curing compound sprayed status into grayscale and perform image clustering for analysis. The main function of the program is to convert the original image into grayscale, and the user can define the section to be analyzed. The image analysis results can be viewed as grayscale images, histograms, and tables, and the analysis data can be saved in a CSV file format.

**Figure 8:**
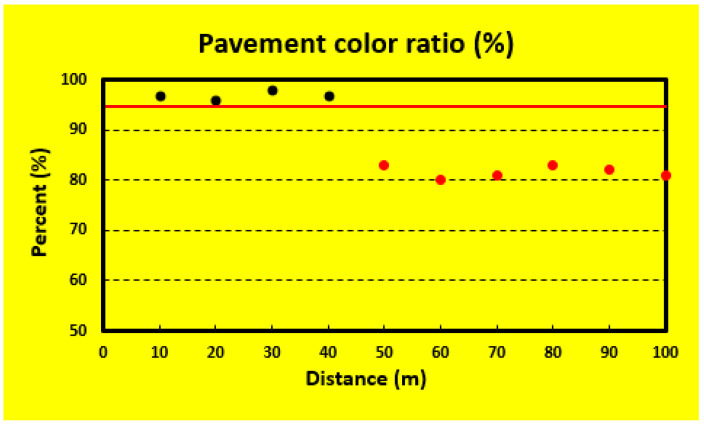
Evaluation results of curing compound sprayed status.

As shown in Figure 8, using the developed program, a graph of the evaluation result for the sprayed status of the curing compound in the analysis section is shown to the user in real time. When the curing compound sprayed status is evaluated as poor below a designated percentage of a reference value, the color of the user screen is changed to yellow, and an alarm is set to sound at the same time.

### 3.3. Determination of Grayscale Reference of Curing Compound Sprayed Status

**Figure 9:**
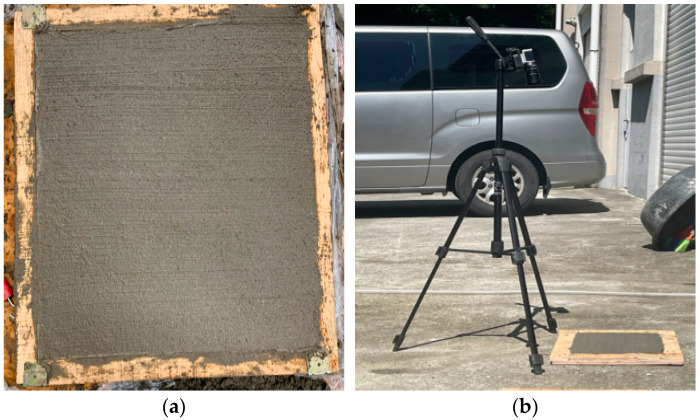
Experiment of grayscale range determination: (**a**) concrete specimen and (**b**) experiment setup.

We performed laboratory experiments to select the reference grayscale range required for the analysis of the curing compound sprayed status. We conducted the experiments by fabricating concrete specimens as shown in Figure 9a in order to proceed similarly to the concrete pavement in the field. Similar to image acquisition at the concrete pavement construction site, the experiments were performed by setting the image sensor to a certain height and angle using a cradle as shown in Figure 9b. The curing compound was sprayed by dividing the curing compound spraying section into 50%, 66%, and 100% on the fabricated concrete specimens, and we conducted the experiments on sunny and cloudy days, respectively, to analyze the effect on environmental conditions.

**Figure 10:**
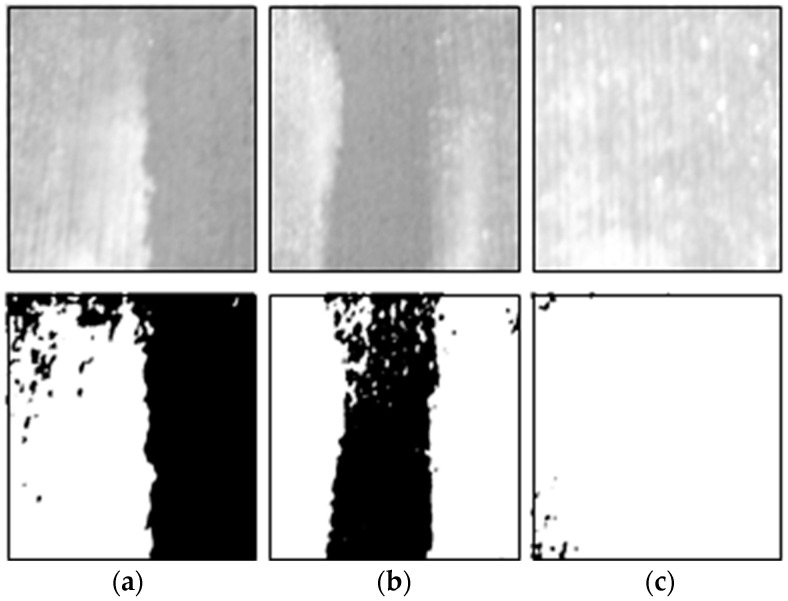
Sprayed status analysis on sunny day: (**a**) sprayed area of 50%; (**b**) sprayed area of 66%; and (**c**) sprayed area of 100%.

**Figure 11:**
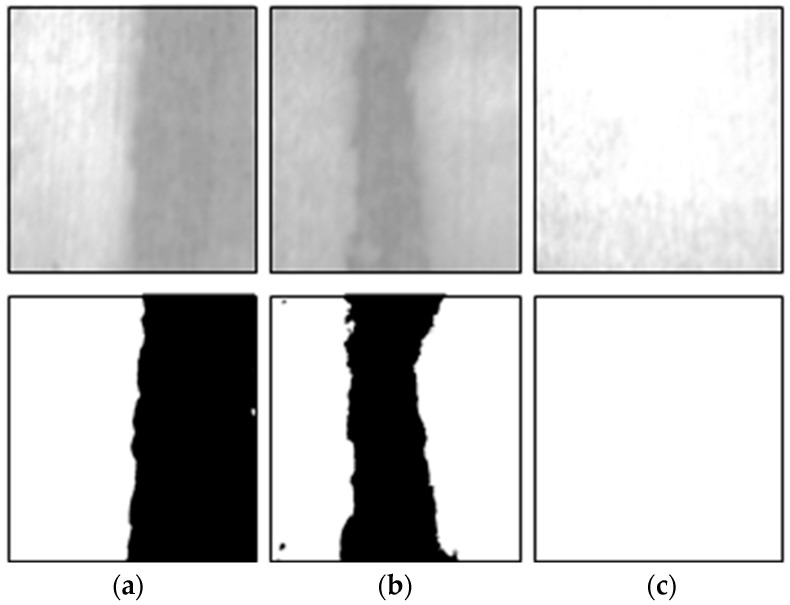
Sprayed status analysis on cloudy day: (**a**) sprayed area of 50%; (**b**) sprayed area of 66%; and (**c**) sprayed area of 100%.

Figure 10 and Figure 11 show the images analyzed for the curing compound sprayed status on sunny and cloudy days, respectively, using the developed program. In the analyzed images, the area where the curing compound is sprayed is displayed in white with a grayscale value of 255, and the concrete surface where the curing compound is not sprayed is displayed in black with a grayscale value of 0. For this analysis, a reference value of grayscale must be set; all areas having a value above the reference value are evaluated as appropriate for spraying the curing compound, and all areas having values below the reference value are evaluated as insufficient spraying of the curing compound.

Table 4 and Table 5 show the results of the experiment for selecting the grayscale reference value for the curing compound sprayed status on sunny and cloudy days, respectively. In sunny weather, the grayscale value range representing the sprayed section of the curing compound was generally over 185, and in cloudy weather, it was generally analyzed to be over 170. Therefore, it is proposed to use a grayscale value of 185 in sunny weather and 170 in cloudy weather, which is used as a reference value when analyzing the curing compound sprayed status. However, these criteria are for reference only to users, and depending on the construction site conditions and environmental conditions, users can change and input a new grayscale reference value defined as the criterion for the appropriateness of the curing compound sprayed status into the program.

### 3.4. Algorithm of Curing Compound Sprayed Status Monitoring System

**Figure 12:**
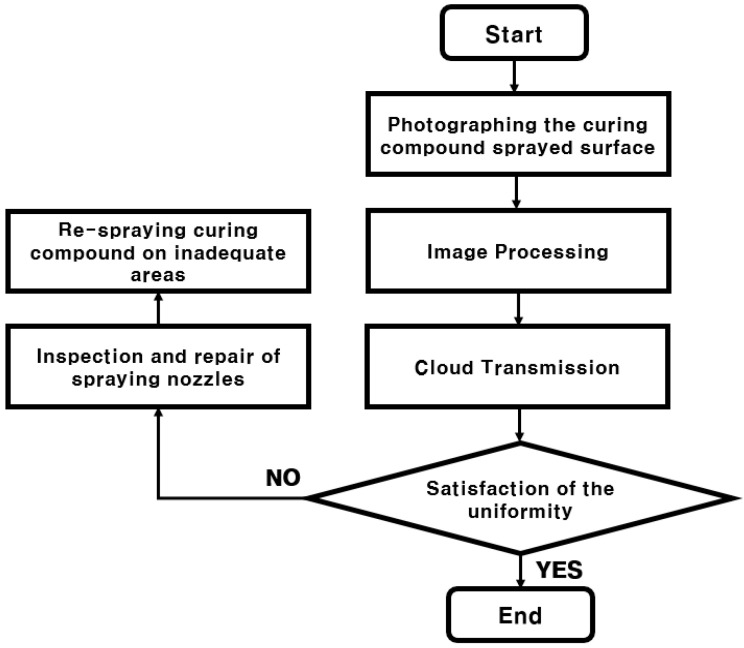
Algorithm of curing compound sprayed status monitoring system.

The algorithm of the curing compound sprayed status monitoring system is shown in Figure 12. First, the sprayed status of the curing compound is photographed using the image acquisition sensor, and at the same time, the image acquisition location is recorded using GPS data. After that, the acquired image is analyzed with an image processing program to evaluate what percentage of the image area satisfies the grayscale reference value or higher.

In the curing compound sprayed status monitoring system, various measured and analyzed values are transmitted and stored by the IoT cloud. These types of data include construction time, spraying equipment speed, equipment’s latitude, longitude and altitude, and ratio above the grayscale reference value of the curing compound sprayed status image. Through the cloud, it is possible to promote quality control by providing measurement and analysis data to construction personnel in real time. If it is evaluated that the curing compound sprayed status is uneven, the spray nozzles of the curing compound spraying equipment are checked and corrected, and the curing compound is re-sprayed in the section where the curing compound is not evenly sprayed.

## 4. Feasibility In Situ Test

**Figure 13:**
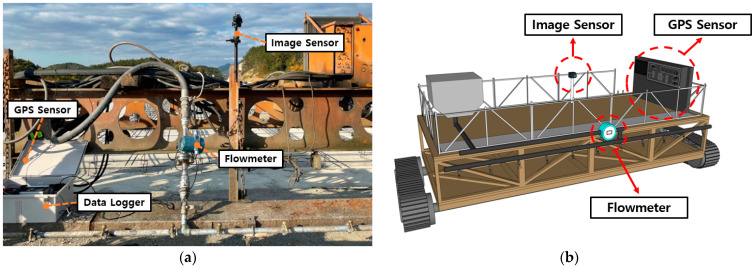
Monitoring system of curing compound application process: (**a**) actual view and (**b**) schematic view.

We conducted two field experiments to confirm the field applicability of the developed IoT-based monitoring system of the curing compound application process. The first field experiment was performed to analyze the applicability of the curing compound spraying amount monitoring system at the construction site of the second ring highway in the Seoul metropolitan area between Yangpyeong and Hwado. The second field experiment was conducted to analyze the applicability of the curing compound sprayed status monitoring system at the construction site of Changnyeong-Milyang Expressway. For the field experiments, as shown in Figure 13, a flow measurement sensor, an image acquisition sensor, and a GPS sensor were installed in the curing compound spraying equipment, and a data logger for measurement, a computer for analysis, transmission equipment for data transmission, and a monitor for real-time observation were also installed. It is noted that there is no minimum internet speed required for the monitoring system to function properly, and the maximum internet speed used in this study was 300 Mbps.

### 4.1. Analysis of Spraying Equipment Location and Speed

**Figure 14:**
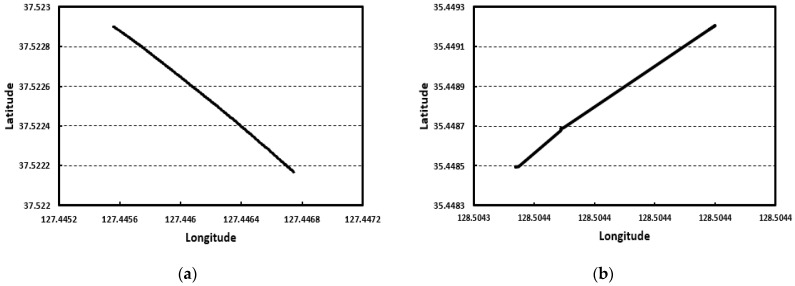
Location of spraying equipment for: (**a**) first experiment and (**b**) second experiment.

Based on the data transmitted to the IoT cloud in the field experiment, the location and speed of the curing compound spraying equipment were analyzed. Figure 14 shows the GPS data analysis results for the curing compound spraying equipment of the first and second field experiments, confirming that the latitude and longitude of the location are properly measured as the spraying equipment moves.

**Figure 15:**
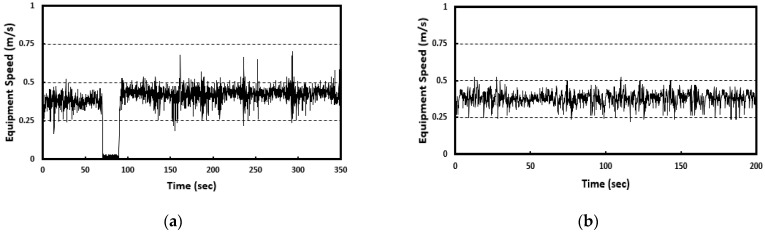
Speed of spraying equipment for: (**a**) first experiment and (**b**) second experiment.

The speed of the curing compound spraying equipment is analyzed as shown in Figure 15 using the relationship between GPS data and time for the spraying equipment. In the figure, the section where the speed is 0 means the state where the curing compound spraying equipment is temporarily stopped, and it can be seen that the curing compound spraying operation is performed at a speed of about 0.4 m/s.

### 4.2. Analysis of Curing Compound Spraying Amount

**Figure 16:**
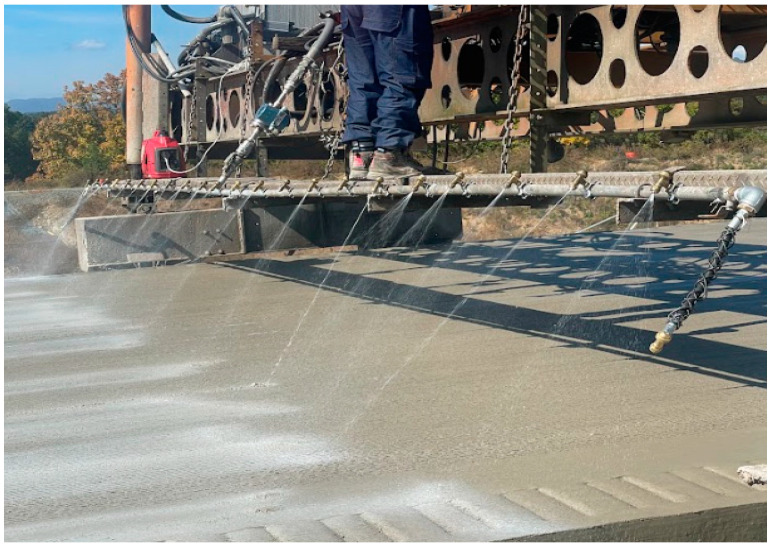
Curing compound spraying process.

In the first field experiment, an analysis of the curing compound spraying amount was conducted at an extension of about 250 m in the concrete pavement construction section. Figure 16 shows the scene where the curing compound spraying equipment sprays the curing compound.

**Figure 17:**
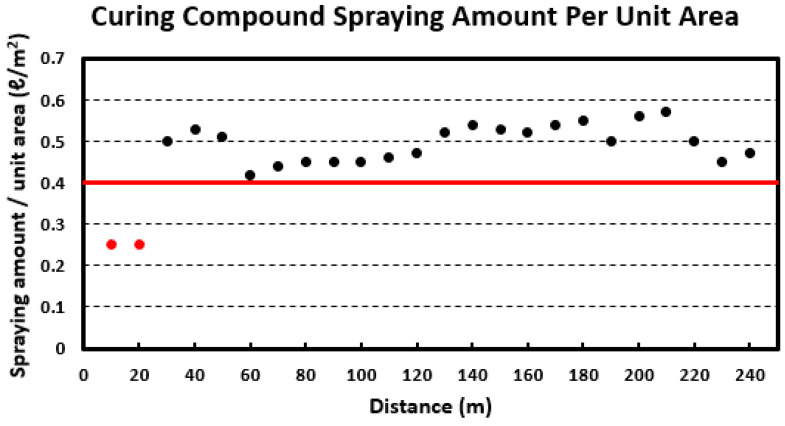
Curing compound spraying amount per unit area.

The analysis result of the curing compound spraying amount per unit area is shown in Figure 17 calculated by considering the curing compound spraying amount and moving speed and distance of the curing compound spraying equipment. The criterion for judging whether the amount of curing compound is properly applied is set at 0.4 L/m^2^, which is the minimum standard for the application amount of curing compound for concrete pavement in Korea [1,2]. As can be seen in the figure, it is confirmed that most of the sections meet the criterion for the amount of curing compound to be sprayed. However, in the 0~20 m section, the curing compound spraying amount standard is not satisfied. This section is the initial section of the curing compound spraying operation and is judged to be the result of the equipment starting to move while the curing compound is not completely filled in the pipe of the curing compound spraying equipment. In this field experiment, approximately 92% of pavement sections met the standard.

### 4.3. Analysis of Curing Compound Sprayed Status

**Figure 18:**
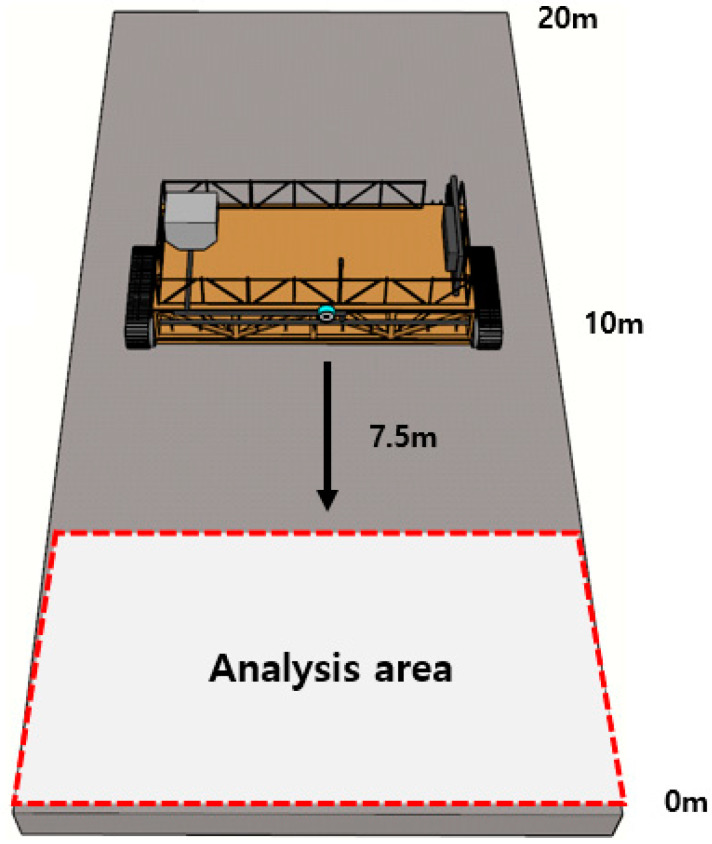
Analysis method of curing compound sprayed status.

In the second field experiment, an analysis of the curing compound sprayed status was performed at an extension of about 70 m in the concrete pavement construction section. As for the method of analyzing the curing compound sprayed status, as shown in Figure 18, taking into account the height of the curing compound spraying equipment and the angle of view of the image acquisition sensor, a section 7.5 m away from the image acquisition sensor was photographed once every 10 m for analysis.

Since the weather was clear during the field experiment, the grayscale standard range of the curing compound sprayed status was selected as 185 or higher. If this standard range was satisfied with 95% or higher, it was set to judge that the curing compound sprayed status was acceptable. For the experiment, firstly, the amount of spraying from each spray nozzle was changed so that the curing compound sprayed status was not uniform. And then, the experiment was performed a second time by re-spraying the curing compound after maintaining the spray nozzle so that the curing compound sprayed status was more uniform.

**Figure 19:**
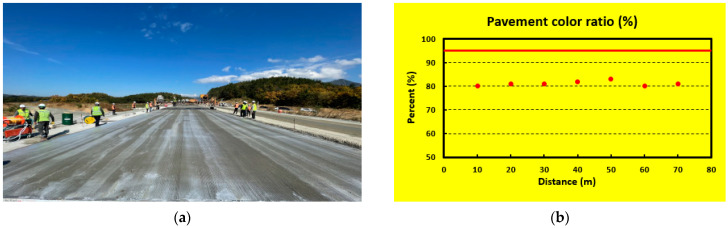
Experiment for the first time: (**a**) scene of sprayed status and (**b**) analysis result.

**Figure 20:**
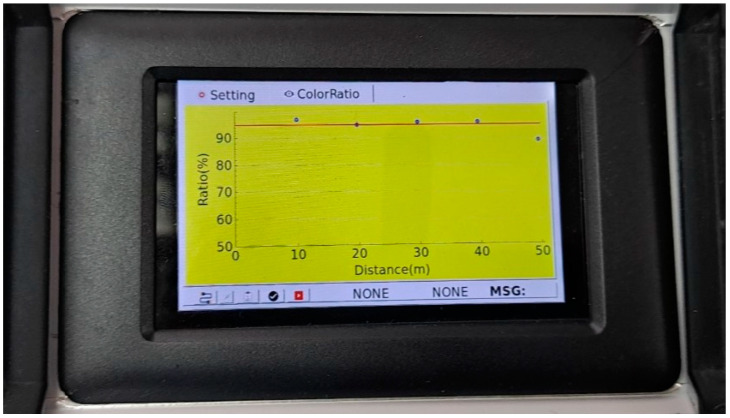
Screen example of curing compound sprayed status monitoring system when the standard is not satisfied.

Figure 19a shows the scene of the sprayed status after spraying the curing compound for the first time, and it can be seen that the curing compound sprayed status is poor. As shown in Figure 19b, the analysis result of the curing compound sprayed status did not satisfy the grayscale standard range at all the sections, so the user’s screen was changed to yellow, and an alarm was immediately delivered to the user. An actual screen example of the curing compound sprayed status monitoring system is shown in Figure 20 when the latest monitored section does not satisfy the standard. In this case, the screen is changed to yellow to warn the operator as mentioned previously.

**Figure 21:**
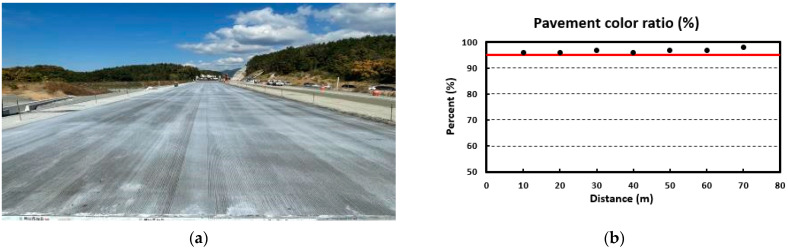
Experiment a second time: (**a**) scene of sprayed status and (**b**) analysis result.

**Figure 22:**
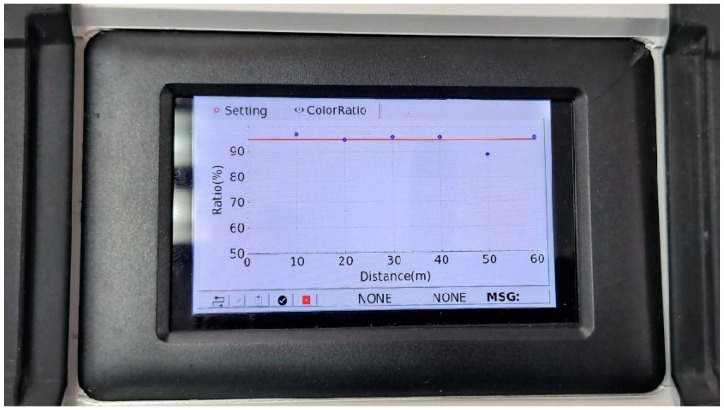
Screen example of curing compound sprayed status monitoring system when the standard is satisfied.

After that, the nozzles of the curing compound spraying equipment were maintained, and the curing compound re-spray work was performed. Figure 21a shows the curing compound sprayed status after respraying the curing compound, and Figure 21b shows the analysis result in which the curing compound sprayed status is good in all sections. An actual screen example of the curing compound sprayed status monitoring system is shown in Figure 22 when the latest monitored section satisfies the standard. In this case, the screen was not changed to yellow as mentioned previously.

## 5. Summary and Conclusions

We conduct this research for the purpose of developing a real-time monitoring system for curing compound spraying process on cement concrete pavement based on IoT technology. The monitoring system is configured to monitor both the curing compound spraying amount and the curing compound sprayed status. The curing compound spraying amount monitoring system is to evaluate whether the curing compound spraying amount per unit area is applied in accordance with the construction standard, and the curing compound sprayed status monitoring system is to evaluate whether the curing compound is evenly sprayed on the surface of the concrete pavement. To achieve the goal of this research, we conduct various laboratory and field experiments. As a result, the IoT-technology-based curing compound spraying amount and sprayed status monitoring systems for cement concrete pavement are established. It is expected that excellent curing compound spraying process of cement concrete pavement will be possible using these monitoring systems. The main results of this research are provided as follows:

(1) We conduct a series of laboratory experiments to analyze the accuracy by selecting a flowmeter capable of measuring the curing compound spraying amount during concrete pavement construction and easy to transmit data in real time. As a result of the flow measurement accuracy test using water and curing compound, the accuracy is over 96%. Therefore, it is confirmed that the selected turbine flowmeter can be applied to measure the curing compound spraying amount in the field;
(2) We select an image acquisition sensor for analyzing the curing compound sprayed status. The image sensor is able to interconnect the IP between the sensor and the personal computer, its output value is over 20 fps for the analysis of the sprayed status, and it can utilize gigabit Ethernet to make image processing more efficient;
(3) To evaluate whether the curing compound is sprayed evenly, we develop an image processing program in this study that can convert the image of the curing compound sprayed status into grayscale and perform image clustering for analysis. The main function of the program is to convert the original image into grayscale, and the user can define the section to be analyzed. The image analysis results can be viewed as grayscale images, histograms, and tables, and analysis data can be saved in a CSV file format;
(4) We perform laboratory experiments to select the reference grayscale range required for the analysis of the curing compound sprayed status. As a result, it is proposed to use a grayscale value of 185 in sunny weather and 170 in cloudy weather. However, these criteria are for reference only to users, and depending on the construction site conditions and environmental conditions, users can change and input a new grayscale reference value defined as the criterion for the appropriateness of the curing compound sprayed status into the program;
(5) We develop the algorithms for the curing compound spraying amount monitoring system and for the curing compound sprayed status monitoring system. In the curing compound spraying amount monitoring system, if it is evaluated that the curing compound spraying amount does not meet the standard, the curing compound spraying process can be calibrated in real time by adjusting the curing compound spraying amount or by adjusting the speed of the curing compound spraying equipment. In the curing compound sprayed status monitoring system, if it is evaluated that the curing compound sprayed status is uneven, the spray nozzles of the curing compound spraying equipment are checked and corrected, and the curing compound is re-sprayed in the section where the curing compound is not evenly sprayed;
(6) In the curing compound spraying amount monitoring system and curing compound sprayed status monitoring system, various measured and analyzed values are transmitted to the IoT cloud and stored. These types of data include construction time, spraying equipment speed, equipment’s latitude, longitude and altitude, curing compound spraying rate, curing compound spraying amount, curing compound spraying amount per unit area, and ratio above the grayscale reference value of the curing compound sprayed status image. Through the cloud, it is possible to promote quality control by providing measurement and analysis data to construction personnel in real time;
(7) We conduct the field experiment to confirm the field applicability of the developed IoT-based curing compound spraying amount monitoring system. Based on the data transmitted to the IoT cloud in the field experiment, the location and speed of the curing compound spraying equipment can be properly analyzed as the spraying equipment moves. It is also confirmed that it is possible to evaluate in real time whether the curing compound spraying amount per unit area is appropriate to the standard;
(8) We conduct the field experiment to confirm the field applicability of the developed IoT-based curing compound sprayed status monitoring system. For the experiment, firstly, the amount of spraying from each spray nozzle is changed so that the curing compound sprayed status is not uniform. And then we perform the experiment a second time by re-spraying the curing compound after maintaining the spray nozzle so that the curing compound sprayed status is more uniform. It is confirmed that when the curing compound sprayed status is not uniform and does not satisfy the grayscale standard range, the user’s screen is changed to yellow, and an alarm is immediately delivered to the user. It is also confirmed that when the curing compound sprayed status is uniform, it is properly evaluated.

To implement the developed monitoring systems in actual construction sites, it is necessary to develop a comprehensive guide including how to attach, detach, and maintain the monitoring devices and how to use the monitoring systems. The guide should also include standard values for the curing compound spraying amount and sprayed status when using the monitoring systems. Further research to improve the monitoring systems can be conducted by applying the monitoring systems to construction sites as much as possible.

## Figures and Tables

**Table 1:** Curing compound application rates [1,2,3,4,5,6,7,8,9,10,11,12,13,14,15,16,17,18].

Country/State	Curing Compound Application Rate (L/m^2^)
Korea	0.4~0.5
United Kingdom	0.22
Australia	0.4
Japan	0.1~0.2
Arizona	0.41
Florida	0.27
Illinois	0.32
Kentucky	0.34
Louisiana	0.41
Michigan	0.36
Mississippi	0.27
New York	0.27
North Carolina	0.27
Texas	0.46
Utah	0.41
Virginia	0.27~0.41
Washington	0.27

**Table 2:** Accuracy analysis of flowmeter using water.

Case	Measured Flow Speed (L/s)	Calculated Volume of Water (L)	Measured Volume of Water in Tank (L)	Accuracy (%)
1	3.62	36.2	37.5	96.5
2	2.53	25.3	26.2	96.6
3	1.51	15.1	15.7	96.2
4	0.79	7.9	7.6	96.1
Average accuracy (%)				96.4

**Table 3:** Accuracy analysis of flowmeter using curing compound.

Case	Measured Flow Speed (L/s)	Calculated Volume of Curing Compound (L)	Measured Weight of Curing Compound in Tank (kg)	Converted Volume of Curing Compound (L)	Accuracy (%)
1	3.64	36.4	31.8	37.0	98.4
2	2.50	25.0	22.3	25.9	96.4
3	1.54	15.4	13.6	15.8	97.4
4	0.78	7.8	7.1	8.3	94.5
Average accuracy (%)					96.7

**Table 4:** Grayscale values during sunny weather.

No.	Area	Specimen of Sprayed Area of 50%	Specimen of Sprayed Area of 66%	Specimen of Sprayed Area of 100%
1	Not sprayed concrete surface	0~190	0~190	0~190
	Curing compound sprayedconcrete surface	191~255	191~255	191~255
2	Not sprayed concrete surface	0~184	0~181	0~184
	Curing compound sprayedconcrete surface	185~255	182~255	185~255
3	Not sprayed concrete surface	0~190	0~190	0~190
	Curing compound sprayedconcrete surface	191~255	191~255	191~255
4	Not sprayed concrete surface	0~177	0~185	0~184
	Curing compound sprayedconcrete surface	178~255	186~255	185~255

**Table 5:** Grayscale values during cloudy weather.

No.	Area	Specimen of Sprayed Area of 50%	Specimen of Sprayed Area of 66%	Specimen of Sprayed Area of 100%
1	Not sprayed concrete surface	0~176	0~174	0~172
	Curing compound sprayedconcrete surface	177~255	175~255	173~255
2	Not sprayed concrete surface	0~174	0~175	0~176
	Curing compound sprayedconcrete surface	175~255	176~255	177~255
3	Not sprayed concrete surface	0~168	0~167	0~168
	Curing compound sprayedconcrete surface	169~255	168~255	169~255

## Data Availability

Not applicable.
